# Associations between Serum Iron Indices and Self-Assessed Multiple Intelligence Scores among Adolescents in Riyadh, Saudi Arabia

**DOI:** 10.3390/biomedicines12071578

**Published:** 2024-07-16

**Authors:** Hibah A. Farhan, Fatimah A. A. Al-Ghannam, Kaiser Wani, Malak N. K. Khattak, Abdullah M. Alnaami, Mona G. Alharbi, Abir A. Alamro, Shaun Sabico, Nasser M. Al-Daghri

**Affiliations:** Biochemistry Department, College of Science, King Saud University, Riyadh 11451, Saudi Arabia

**Keywords:** multiple intelligence, McKenzie questionnaire, iron, transferrin, adolescents, nutritional status

## Abstract

Micronutrient deficiencies, including iron deficiency, are linked to different cognitive impairments and sensory functions. However, whether circulating iron levels affect self-assessed multiple intelligence (MI) scores in adolescents remains uninvestigated. This study aimed to investigate associations between serum iron levels and self-assessed MI scores in adolescents in Riyadh, Saudi Arabia. Recruiting 434 Saudi adolescents (174 boys and 260 girls, aged 12–17), we administered the McKenzie questionnaire to assess MI across nine categories. Anthropometrics and fasting blood samples were collected to measure circulating iron and transferrin levels. Total iron-binding capacity (TIBC) and transferrin saturation (TSAT) levels were calculated. Notably, girls exhibited significantly higher MI scores in the interactive domain than boys (age and BMI-adjusted OR = 1.36, 95% confidence interval = 1.07–1.73, *p* = 0.01). No significant correlations were observed between serum iron and MI. However, normal TSAT levels (TSAT > 20%) corresponded with higher age and BMI-adjusted odds of MI scores in the musical (OR = 1.59, 95%CI = 1.1–2.2, *p* = 0.006), linguistic (1.57, 1.1–2.3, *p* = 0.016), kinesthetic (1.48, 1.1–2.1, *p* = 0.024), spatial (1.45, 1.1–2.1, *p* = 0.03), and existential (1.56, 1.1–2.1, *p* = 0.01) categories compared to ones with lower TSAT levels (TSAT ≤ 20%), only in boys. In conclusion, serum iron levels may not directly influence MI domains in adolescents in Riyadh, Saudi Arabia; however, lower TSAT levels, indicative of iron-deficiency anemia, may influence MI, only in boys, indicating a possible relationship between iron metabolism and cognitive functions.

## 1. Introduction

Deficiencies in micronutrients, particularly iron, are intricately tied to diverse cognitive impairments and can potentially induce enduring behavioral alterations [1]. Cognition, defined as the mental processes involved in acquiring knowledge, encompasses perception, reasoning, creativity, problem-solving, and possibly intuition, significantly influencing quality of life [2]. The role of iron in cognitive functions has been suggested in some early studies [3,4], linking iron deficiencies to alterations in psychomotor development and cognitive functions. Moreover, emotional and behavioral alterations have also been linked with iron deficiency [5]. Studies investigating the relationship between iron and cognitive function have been mostly focused on children [6,7], while its role in the age of adolescence has not been studied much.

Iron deficiency, particularly when it occurs during critical periods of brain development, has profound and lasting effects on cognitive outcomes [8]. During these sensitive developmental stages, iron is crucial for processes such as myelination, neurotransmitter synthesis, and overall brain growth [9,10,11]. The duration and severity of iron deficiency also play a critical role, with prolonged periods of deficiency adding to the severity of cognitive impairments [12]. Research has shown that even after iron levels are corrected, cognitive deficits may persist, indicating the importance of early detection and intervention [13]. In Saudi Arabia, iron deficiency is prevalent among adolescents, with roots often traced back to early childhood [14]. Factors such as dietary habits, socioeconomic status, and genetic predispositions contribute to this high prevalence [15,16]. The early onset of iron deficiency in this population stresses the need for continuous monitoring and preventive measures from infancy to adolescence to mitigate its long-term cognitive impact.

Among the qualities of cognition is intelligence, and while many definitions of intelligence traditionally emphasize academic achievement [17], it is recognized that certain facets, such as problem-solving, elude comprehensive assessment through standard short-answer tests. Drawing on psychometric and neuropsychological insights, Harvard psychologist Howard Gardner proposed the concept of multiple intelligence (MI) [18] and theorized that individuals possess multiple intelligences, such as musical, interpersonal, spatial–visual, and linguistic intelligences. Linguistic, logical, spatial, kinesthetic, interpersonal, intrapersonal, naturalistic, musical, and existential intelligences are all important aspects of human intelligence, each requiring specific skills and abilities to excel in various aspects of life.

To the best of our knowledge, no studies have investigated the association between circulating levels of iron and transferrin and different facets of MI in adolescents. Building on these findings and considering the role of iron in cognitive function and brain development, we hypothesized an association between serum iron levels and self-assessed MI scores in adolescents in Riyadh, Saudi Arabia. This study aimed to contribute insights into the complex interplay between serum iron levels and self-assessed MI scores in the adolescent population.

## 2. Materials and Methods

### 2.1. Study Participants

**Figure 1:**
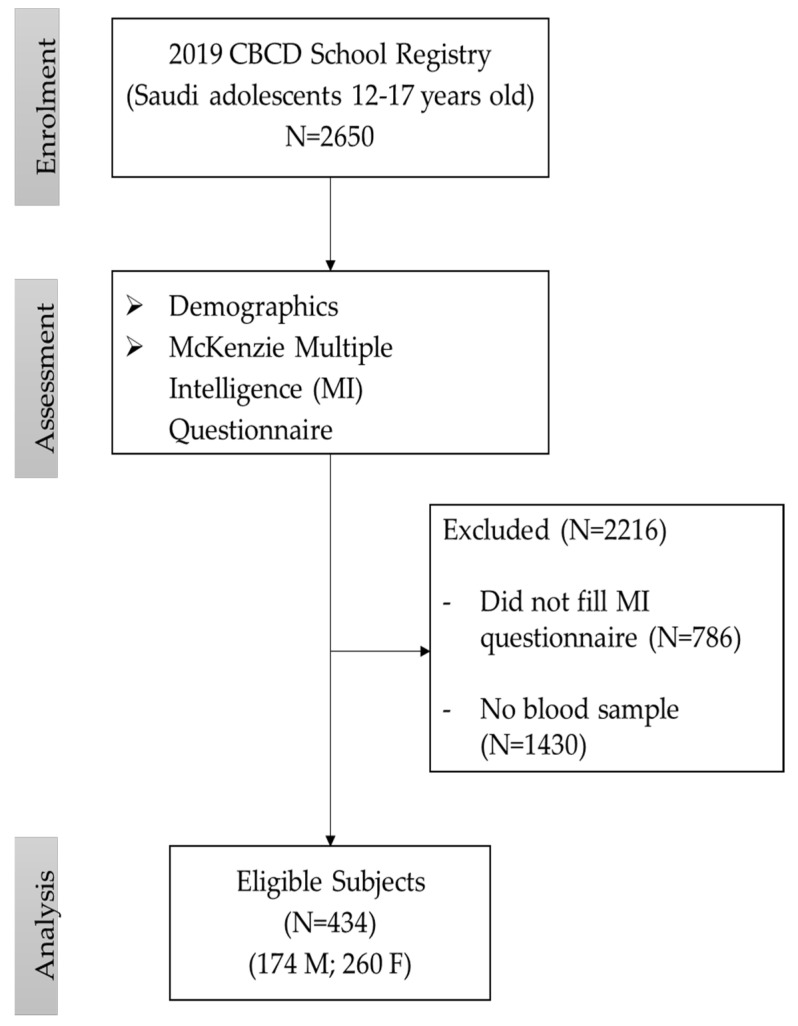
Flowchart of study participants.

In the present study, the participants were randomly selected from the database of the “School Registry-2019” at the Chair for Biomarkers of Chronic Diseases (CBCD) in King Saud University, Riyadh, Saudi Arabia. In brief, the registry includes data from Saudi adolescents recruited under a lifestyle change interventional counselling program conducted by the CBCD and the Saudi Charitable Association of Diabetes (SCAD) across 60 high schools and preparatory year schools around Riyadh, Saudi Arabia [19,20]. The purpose of this program was to raise awareness of the increasing prevalence of obesity, diabetes, and metabolic syndrome among Saudi Arabian youth [21]. The inclusion criteria were Saudi adolescents, aged 12–17 years, without chronic diseases like heart, kidney, and liver diseases, psychiatric conditions, and/or who were not taking medication used for such diseases. Out of the 2650 adolescents in the registry, 1864 consented to participate. The flowchart of the participants recruited for this study has been provided in Figure 1. This study was approved by the Institutional Review Board (IRB), Health Sciences Colleges Research on Human Subjects, King Saud University-College of Medicine (approval number E-23-8231).

### 2.2. Questionnaire Data

Two questionnaires were administered and answered by each participant in this study. The first one collected clinical information about the participants such as age, gender, weight (in kilograms), height (in centimeters), waist circumference, hip circumference (in centimeters), individual medical history, etc. Blood pressure information was collected by the recruiting personnel using digital portable blood pressure monitors as an average of two readings recorded with a 15 min interval. One more questionnaire was administered: the McKenzie Multiple Intelligence (MI) questionnaire [22,23], to elicit the MI scores of the participants. The questionnaire used in our study is well-suited for assessing MI preferences across age ranges. The McKenzie MI questionnaire, adapted from Gardner’s theory [18] of multiple intelligences, is not a traditional test with right or wrong answers; instead, it serves as a reflective tool to capture a snapshot of an individual’s perceived MI preferences at a given point in time. The questionnaire’s design includes 90 items (Supplementary File S1), featuring simple statements that respondents answer on a Likert scale to indicate their level of agreement with each statement, making it accessible and relevant to both younger and older adolescents. The responses to the questions were collected under standardized conditions and the participants were instructed clearly to reduce the likelihood of exaggerated self-assessment. Supplementary Figure S1 summarizes the 9 facets and 3 domains of MI proposed by Gardener [18].

### 2.3. Biochemical Parameters

A fasting blood sample was taken from each participant for the biochemical analysis and the procedure was facilitated by well-trained research nurses assigned for this project. Blood samples (approximately 5 ccs) were immediately transferred to a non-heparinized tube for centrifugation and the serum samples obtained were transferred to a pre-labelled tube, stored on ice, and delivered to the CBCD, where routine biochemical analysis like glucose and lipid profiles, was performed on the same day or stored in at −80 °C in a freezer before further analysis was conducted. The circulating glucose levels and lipid profiles (total cholesterol, HDL cholesterol, and triglycerides) were analyzed using an automated biochemical analyzer (Konelab, Espoo, Finland) using routine bioassay kits (catalogue #981379, 981812, 981823, and 981301, respectively from Thermo Fischer Scientific, Vantaa, Finland). The circulating transferrin levels were estimated by enzyme-linked immunosorbent assay (ELISA) using an assay kit (catalogue #EHTF, Invitrogen, ThermoFisher Scientific, Carlsbad, CA, USA). The sensitivity of the kit was 1 ng/mL, and the inter- and intraassay CVs were <12% and <10%, respectively. Serum iron levels were quantified by biochemical analyzer Konelab using a bioassay kit (catalogue #981236), which utilizes guanidine buffer, and ascorbic acid to release iron from its carrier protein in the samples and form a colored complex with Ferene in the assay reagent, whose intensity was measured at 600 nm. The sensitivity of the bioassay kit was 0.1 µmol/L and the imprecision rate was <3% of the total CV. Total iron-binding capacity (TIBC) and transferrin saturation (TSAT) levels were calculated using the values of circulating iron and transferrin levels as below [24,25]:

Transferrin (mg/dL) = 0.8 × TIBC (mg/dL) − 43

TIBC (mg/dL) = (Transferrin (mg/dL) + 43) ÷ 0.8

And TSAT (%) = (Iron (mg/dL) ÷ TIBC (mg/dL)) × 100

### 2.4. Power Calculation and Data Analysis

Data generated from the questionnaires (MI, anthropometrics, and the results from the blood sample analysis) were compiled in a Microsoft Excel sheet using a unique ID for each participant. The data were analyzed using the Statistical Package for the Social Sciences software (SPSS 16.0; SPSS Inc., Chicago, IL, USA). We used the Kolmogorov–Smirnov test to ensure that our data were normally distributed. Normally distributed data were presented as means and standard deviations (SDs) and non-normal data were presented as medians (1st and 3rd quartiles). The average values and the standard deviations for each parameter were compared between genders and the differences were tested using student’s *t*-tests. A correlation analysis was performed between serum iron levels, other parameters, and the MI scores to determine associations. Multivariate linear regression was then performed to present the changes in MI scores for a unit change in iron levels, and the results were adjusted for covariates such as age and BMI, and the data were presented as β-coefficient and its 95% confidence intervals. The participants were then divided into those with TSAT levels ≤ 20%, which indicates iron deficiency [26], and those with TSAT levels > 20%, and a multivariate regression analysis was conducted with TSAT levels as the dependent variable and the MI scores as the independent variables to determine the odds of MI scores in the group with TSAT > 20% vs. TSAT ≤ 20%, and the analysis was reported. Microsoft Excel was used to plot the graphs.

## 3. Results

### 3.1. Clinical Characteristics and MI Scores in Study Participants

The anthropometric, clinical characteristics, and MI scores of the study participants are presented in Table 1. A total of 434 Saudi adolescents [174 boys (40.1%) and 260 girls (59.9%)] were included in this study. The mean age of the participants was 14.7 ± 1.5, with boys slightly older than girls (14.9 vs. 14.5 years, *p* = 0.02). WHR was significantly higher in boys than girls (0.86 ± 0.11 vs. 0.79 ± 0.11, *p* < 0.001). The mean glucose and triglyceride levels in the boys were higher compared to the girls (*p* = 0.008 and *p* = 0.02, respectively). Serum iron and transferrin were higher in girls than boys (17.86 μmol/L vs. 15.39 μmol/L, *p* < 0.001 for iron and 3.10 mg/mL vs. 2.39 mg/mL, *p* = 0.002 for transferrin). Serum TIBC was higher in boys compared to girls (441.34 µg/dL vs. 352.83 µg/dL). However, TSAT levels were comparable between the genders and TSAT levels ≤ 20%, indicative of iron deficiency, was 36.8% and 38.7% in boys and girls, respectively.

The MI scores were also analyzed between the genders. Girls showed significantly higher mathematical (*p* < 0.001), social (*p* = 0.001), kinesthetic (*p* = 0.02), interactive (*p* = 0.01), interpersonal (*p* = 0.06), and introspective MI’s (*p* = 0.03) than boys. The average total MI score in girls was higher than in boys (2.59 ± 0.7 vs. 2.41 ± 0.9, *p* = 0.02).

### 3.2. Comparison of MI Scores between Genders

The MI scores obtained for each of the facets were analyzed using logistic regression models, taking the scores obtained in boys as the reference, and the results are presented in Table 2. The age and BMI-adjusted ORs of MI scores were significantly greater in girls for the logical, social, kinesthetic, interpersonal, and introspective categories. The average total MI scores for girls were thus significantly greater compared to boys (age and BMI-adjusted OR = 1.31 (95% CI 1.02–1.67), *p* = 0.035).

**Figure 2:**
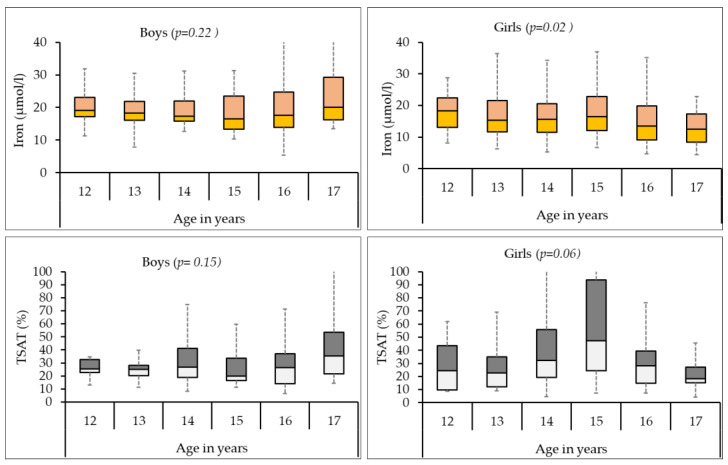
The age-wise distribution of serum iron and transferrin levels in boys and girls. The *x*-axis represents age in years, while the *y*-axis represents serum iron or transferrin saturation levels. The graph shows a negative correlation between age and iron levels in girls, indicating that even in adolescence, as girls get older, their circulating iron levels tend to decrease.

Figure 2 shows the age-wise distribution of serum iron and transferrin levels in boys and girls. While in boys, both iron and transferrin levels did not show any age-wise trend, in girls, we observed a constant decrease in average serum iron levels with age (*p* = 0.02), while transferrin saturation levels increased till age 15 after which they decreased (*p* = 0.06).

### 3.3. Correlation Analysis of Serum Iron and Transferrin Saturation Levels with Other Parameters

Table 3 presents the correlation coefficients of association between iron or transferrin saturation levels and the other measured parameters. A significant negative correlation was observed between iron levels and age in girls (r = −0.22, *p* < 0.01), while iron levels were not correlated with age in boys. BMI also showed a significant inverse correlation with serum iron levels in girls (r = −0.14, *p* < 0.05), while the negative correlation in boys was not significant. Similarly, an overall negative correlation of serum iron levels was observed with waist and hip circumferences in girls (r = −0.19, *p* < 0.001 and r = −0.23, *p* < 0.001 for waist and hip circumferences, respectively). With circulating lipid levels, serum iron levels showed an overall positive correlation (r = 0.16, *p* < 0.001 and r = 0.28, *p* < 0.001 for cholesterol and triglycerides, respectively). With transferrin saturation levels, an overall positive correlation was seen with waist (r = 0.19, *p* < 0.05), hip circumference (r = 0.20, *p* < 0.05), systolic and diastolic BP (r = 0.19, *p* < 0.05), and triglycerides (r = 0.18, *p* < 0.05) in boys only.

When an association with different MI scores was checked, serum iron levels showed no significant overall or gender-specific correlations. However, with transferrin saturation levels, a gender-specific correlation was seen with linguistic (r = 0.18, *p* < 0.05), kinesthetic (r = 0.21, *p* < 0.05), existential (r = 0.22, *p* < 0.05), interactive (r = 0.19, *p* < 0.05), and introspective (r = 0.18, *p* < 0.05) MIs only in boys.

### 3.4. Association of Serum Iron Levels with MI Scores for All Participants

We used linear regression models where the dependent variable was the MI score and the independent variable was the serum iron level, and the analysis is presented in Table 4. The analysis showed that serum iron levels had no association with any of the MI groups in both the unadjusted and the adjusted models. The data for all participants were used for the regression analysis without dividing into genders, since no correlation with iron levels was seen for MI scores in either of the sexes (Table 3).

### 3.5. Association of Serum Iron Levels with MI Scores According to TSAT Levels

The participants were divided into those with transferrin saturation levels ≤ 20% and >20%, and a regression analysis was performed to check the odds of the MI scores in the group with TSAT levels > 20% compared to the group with ≤20%; the odds ratio were then adjusted for age and BMI. The results of the regression analysis are presented in Table 5. The odds of the MI scores in all nine MI categories and the three MI domains were comparable between the two groups when the data were analyzed for all participants; however, stratification by gender revealed age and BMI-adjusted odds of higher MI scores for musical (*p* = 0.006), linguistic (*p* = 0.016), kinesthetic (*p* = 0.02), spatial (*p* = 0.03), and existential (*p* = 0.012) scores in those with TSAT > 20% vs. TSAT ≤ 20%, only in boys.

## 4. Discussion

In this study, we aimed to explore potential associations between circulating iron levels and self-assessed MI scores, considering the recognized links between micronutrient deficiencies, particularly iron, and cognitive impairments in attention span and sensory perception functions. We explored this association using a validated tool of the 90-item McKenzie questionnaire to assess the nine facets of MI in Saudi adolescents. The main findings in this study included a gender-specific assessment of MI scores wherein girls scored significantly higher in the interactive and introspective domains compared to boys. Also, we found that the circulating levels of the micronutrient iron in adolescent girls decreased significantly with age and BMI. However, as far as the main objective of this study is concerned, we found no significant association between the circulating iron levels and different facets of MI scores, either in the bivariate models or in regression models when adjusted for age and BMI. Normal TSAT levels (TSAT > 20%) were, however, associated with higher odds of MI scores compared with lower TSAT levels (TSAT ≤ 20% indicative of iron deficiency), only in boys.

Our study observed notable gender differences in MI scores, with girls reporting higher scores, particularly in the interactive and introspective domains of MI. These differences may indicate potential variations in self-perceived cognitive abilities between boys and girls, though caution is required due to the self-reported nature of the data. Some earlier studies found significant gender effects in certain types of intelligence, and our study findings align with their observations. A study by Buczylowska et al. [27], for example, found higher MI scores achieved by girls compared to boys in early childhood and the differences became negligible during development. The authors explained that this may be because girls tend to mature earlier in regard to cognitive abilities. In some other reports also [28,29], higher scores, especially in the interactive and introspective domains, were observed in females compared to males. These higher scores may be attributed to socialization processes that emphasize communication and emotional intelligence in girls from a young age [30,31]. Furthermore, studies such as [32] have highlighted that females often exhibit enhanced interpersonal skills, contributing to higher scores in interactive domains.

Reports have proposed that hormonal and neurological variations between the genders may influence cognitive strengths, with females showing advantages in certain cognitive domains [33,34]. Many reports in self-estimated intelligence, however, suggested that males systematically self-report higher estimates than females, a phenomenon termed as the “male hubris, female humility” effect [35,36], while in some other studies, no significant gender differences in overall MI scores were reported [37,38]. It is important to note that the results varied depending on the specific MI test used and the cultural context of these studies. The observed gender disparities in our study, however, highlight the need for continued exploration into the intricate interplay between gender and MI scores, offering valuable insights for educational and psychological research.

This study observed a significant inverse correlation between age and iron levels in girls, suggesting that as age increases within the adolescent period, serum iron levels tend to decrease in girls. This observation may have various implications related to the physiological changes, growth, and nutritional needs during adolescence, and correspond with the findings reported in some earlier studies [39,40]. The decrease in circulating iron levels in girls with age may occur through several potential mechanisms. The period of adolescence is marked by rapid growth and development, particularly during puberty, and increased demands for iron to support the expansion of blood volume and muscle mass may contribute to the observed decline in serum iron levels [41]. Menstruating teenage females often lose iron through menstrual blood, and as they grow through puberty, the frequency and volume of menstrual cycles may rise, impacting iron levels even more [42]. Additionally, a change in dietary choices or an insufficient consumption of iron-rich foods throughout this period may exacerbate decreased blood iron levels [43], which indicates that iron levels in teenage females change with age, with different life phases and reproductive variables impacting them.

The observed decrease in serum iron levels with increasing BMI in adolescent girls found in this study was also reported by earlier studies [44,45], suggesting a complex interplay between iron metabolism and body composition. The role of adipose-related inflammatory cytokines in hindering iron absorption and utilization [46] may be one possible mechanism explaining this decrease in serum iron levels with BMI. Higher BMIs may also be associated with increased levels of hepcidin, a key regulator of iron metabolism that can limit iron absorption and release from stores, leading to lower serum iron levels [47].

The lack of a significant correlation between serum iron levels and multiple intelligence scores, in univariate as well as age and BMI-adjusted models, suggests that, in the context of the study population, serum iron status may not be a predominant factor influencing various facets of intelligence. Intelligence is a multifaceted trait influenced by various genetic, environmental, educational experiences, socio-economic background, and other cognitive factors. Iron, while crucial for cognitive function, may not be a significant determinant of diverse intelligence facets [47]. Furthermore, cognitive abilities are highly individualized, and the impact of iron levels on intelligence may vary among individuals, while genetic and environmental factors may play substantial roles [48,49]. These, together with results like ours, suggest that while nutrients like iron play a vital role, intelligence is influenced by a myriad of factors that may not exhibit direct linear correlations in certain contexts.

Contrastingly, some studies support the direct role of iron and iron supplementation in improving the intelligence and cognitive abilities of children. A systemic review and meta-analysis of nine studies from five countries observed that iron supplementation had a positive impact on intelligence test scores; however, no significant effects on attention, short or long-term memory, or school performances were found [50]. Similarly, another meta-analysis of 13 studies of iron supplementation on school children aged 6–12 years concluded that it had a significant positive impact on intelligence, attention, and memory; however, no significant effect on school achievement was observed [51]. The absence of a significant correlation between serum iron levels and MI scores in this study may be influenced by the presence of a threshold effect, where the impact of iron on cognitive outcomes becomes noticeable only below or above specific concentration levels. The concept of a threshold effect in the context of nutrient influence on cognitive function suggests that there may be a certain level or range of the nutrient below or above which cognitive outcomes are unaffected [8].

On further analysis, when individuals were divided into those with TSAT levels > 20% and TSAT levels ≤ 20% (indicative of iron deficiency), higher overall age and BMI-adjusted odds of MI scores in the TSAT > 20% group vs. TSAT ≤ 20% were observed, only in boys. This novel observation presents intriguing insights into the complex relationship between iron metabolism and cognitive functions in adolescents and suggests that transferrin saturation, a key indicator of iron deficiency, may influence the impact of iron on specific cognitive abilities. TSAT, being a dynamic marker, provides valuable information into immediate iron availability for biological processes, which is particularly relevant when exploring associations with cognitive performance and intelligence scores. The observed association between normal TSAT levels (>20%) and higher MI scores in boys may be explained through several biological mechanisms that signify the essential role of iron in cognitive function. Iron is a vital component in numerous neurobiological processes, including myelination, neurotransmitter synthesis, and oxygen transport to the brain, processes that are particularly important for optimal cognitive development and function [9,10,11]. A recent review of 26 cross-sectional and 24 iron-containing interventions concluded that iron status or anemia influenced attention, intelligence, and memory in adolescents [52]. Normal TSAT levels provide enough iron for dopamine production, which may improve cognitive skills associated with the analytical and interactive domains of MI [53]. Adequate iron levels, indicated by normal TSAT levels, also help to maintain white matter integrity, thereby enhancing cognitive functions such as information processing speed and connectivity across different brain regions [54]. Cognitive development and iron metabolism may differ between genders due to hormonal influences, as indicated by our gender-specific associations between TSAT levels and MI scores. Boys, who generally have higher metabolic and physical activity rates, might experience more significant cognitive benefits from bioavailable iron levels due to the increased demand for energy and oxygen transport, which supports enhanced MI scores [55].

Several limitations of this study may affect the interpretation of the results and must be acknowledged. Firstly, the cross-sectional nature of this study limits the causality of the findings and longitudinal studies, especially ones with iron supplementation, may validate and consolidate the findings. Our cross-sectional study, however, offers valuable initial findings regarding the associations between serum iron and TSAT levels and MI indices in Saudi adolescents, which we believe can guide and inform future longitudinal research in this area. In this study, we did not measure serum ferritin levels, which could have yielded the true iron status of the participants. However, the transferrin saturation levels assessed in this study may also be indicative of iron deficiency anemia for TSAT levels ≤ 20%. Self-assessed intelligence scores, as presented in this study, may present response bias. However, the 90-item McKenzie questionnaire, proposed and developed by Harvard psychologist Howard Gardner, is a validated and established tool to assess multiple intelligence scores, and it remains one of the few comprehensive tools available for such assessments, in addition to covering a broad range of intelligence domains that aligns with the MI theory. Also, despite their subjective nature, self-assessment measures provide unique insights into participants’ perceptions of their intelligence across various domains. Furthermore, our study did not account for other factors that can influence iron and transferrin levels, such as dietary factors, nutritional status, inflammation, etc. We would, however, like to point out that, though the data for socioeconomic, dietary, and genetic factors were not individually assessed, this study was conducted in a homogenous population, minimizing the variability that might arise from such differences. Lastly, this study was conducted across multiple schools in Riyadh, KSA, and the findings might not be generalizable to other regions or populations. Future studies, including well-designed clinical trials or large-scale prospective cohorts, may thus be needed in this population to overcome these limitations and provide more robust evidence on the correlation between iron, transferrin, and MI scores.

## 5. Conclusions

To summarize, our study findings indicate that serum iron levels alone may not have a direct effect on various aspects of Multiple Intelligence (MI) among Saudi adolescents. We did, however, observe an interesting association in Saudi adolescents, only among boys, where lower TSAT levels (≤20%)—indicative of iron-deficiency anemia—were associated with lower MI scores in the analytical and interactive domains of MI. These findings signify the importance of considering a comprehensive profile of iron metabolism, including serum iron and related markers like transferrin, to better understand its impact on cognitive function in this population. To more effectively understand the complex relationships between iron metabolism and cognitive functions, future studies need to include broader assessments on nutritional status, genetic factors, and longitudinal designs, along with a comprehensive panel of iron biomarkers, such as ferritin and hepcidin.

## Figures and Tables

**Figure 3:**
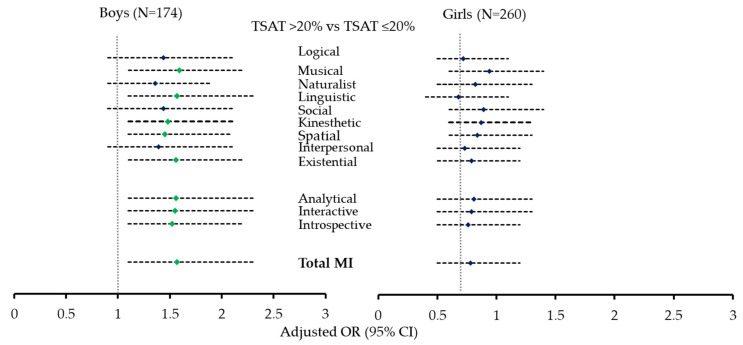
Forest plot representing the age and BMI-adjusted odds ratio for MI scores based on TSAT levels. Significant OR’s are represented by green rhombuses.

**Table 1:** Clinical characteristics and MI scores in the study participants.

Parameters	All	Boys	Girls	*p*-Value
N	434 (100.0)	174 (40.1)	260 (59.9)	
Age (years)	14.7 ± 1.5	14.9 ± 1.5	14.5 ± 1.5	0.02
Weight (kg)	56.3 ± 17.1	61.1 ± 19.7	53.1 ± 14.3	<0.001
BMI (kg/m^2^)	23.3 ± 7.3	23.6 ± 6.4	23.1 ± 7.8	0.49
Waist (cm)	71.1 ± 17.3	69.2 ± 22.7	72.4 ± 12.5	0.06
Hips (cm)	86.6 ± 19.8	79.3 ± 26.5	91.4 ± 11.6	<0.001
WHR	0.82 ± 0.11	0.86 ± 0.11	0.79 ± 0.11	<0.001
Systolic BP (mmHg)	117.9 ± 14.9	118.4 ± 14.5	117.5 ± 15.4	0.52
Diastolic BP (mmHg)	71.4 ± 11.8	66.4 ± 9.7	74.6 ± 11.9	<0.001
Glucose (mmol/L)	5.22 ± 0.6	5.32 ± 0.6	5.16 ± 0.6	0.008
T-Cholesterol (mmol/L)	4.39 ± 0.7	4.33 ± 0.7	4.44 ± 0.7	0.14
HDL-Cholesterol (mmol/L)	1.02 ± 0.2	1.02 ± 0.2	1.01 ± 0.2	0.61
Triglycerides (mmol/L)	0.95 (0.8–1.3)	1.04 (0.8–1.4)	0.91 (0.7–1.2)	0.02
Iron (μmol/L)	16.76 (12.4–21.8)	17.86 (15.4–23.3)	15.39 (11.2–20.8)	<0.001
Transferrin (mg/dL)	301.76 (166.8–377.2)	310.00 (217.3–378.1)	239.30 (66.8–368.4)	0.002
TIBC (µg/dL)	430.96 (262.4–525.3)	441.34 (325.4–526.4)	352.83 (137.3–514.2)	0.002
TSAT (%)	25.17 (16.4–40.9)	25.17 (17.4–38.9)	25.28 (14.9–53.9)	0.646
TSAT (≤20%)	37.7	36.8	38.7	0.771
MI scores (9 categories)				
Logical	2.49 ± 0.9	2.28 ± 0.9	2.63 ± 0.9	<0.001
Musical	2.77 ± 0.9	2.80 ± 1.1	2.75 ± 0.9	0.56
Naturalist	2.63 ± 0.9	2.55 ± 0.9	2.68 ± 0.8	0.13
Linguistic	2.58 ± 0.9	2.50 ± 0.9	2.64 ± 0.8	0.09
Social	2.49 ± 0.9	2.33 ± 0.9	2.60 ± 0.8	0.001
Kinesthetic	2.46 ± 0.9	2.33 ± 0.9	2.54 ± 0.9	0.02
Spatial	2.53 ± 0.9	2.43 ± 0.9	2.60 ± 0.9	0.06
Interpersonal	2.25 ± 0.8	2.11 ± 0.9	2.34 ± 0.8	0.006
Existential	2.45 ± 0.9	2.37 ± 1.0	2.51 ± 0.9	0.133
MI scores (3 domains)				
Analytical	2.63 ± 0.8	2.54 ± 0.9	2.69 ± 0.8	0.08
Interactive	2.51 ± 0.8	2.38 ± 0.9	2.59 ± 0.8	0.01
Introspective	2.41 ± 0.8	2.30 ± 0.9	2.48 ± 0.8	0.03
Total MI	2.52 ± 0.8	2.41 ± 0.9	2.59 ± 0.7	0.02

Note: Data were presented as mean ± standard deviation and median (Q1–Q3) for normal and non-normal continuous variables. The difference between the genders was calculated by appropriate statistical tests according to the nature of the variables. *p* < 0.05 was taken as significant.

**Table 2:** Logistic regression analysis showing MI scores for girls compared to boys.

MI Categories	Boys	Girls			
		Unadjusted		Adjusted with Age and BMI	
		OR (95% CI)	*p*	OR (95% CI)	*p*
Logical	Ref	1.56 (1.25–1.96)	<0.001	1.57 (1.25–1.97)	<0.001
Musical	Ref	0.94 (0.78–1.45)	0.56	0.90 (0.74–1.10)	0.32
Naturalist	Ref	1.18 (0.95–1.47)	0.12	1.17 (0.94–1.46)	0.16
Linguistic	Ref	1.21 (0.96–1.51)	0.09	1.22 (0.97–1.54)	0.09
Social	Ref	1.45 (1.15–1.82)	0.001	1.44 (1.14–1.82)	0.002
Kinesthetic	Ref	1.27 (1.03–1.57)	0.022	1.27 (1.03–1.58)	0.03
Spatial	Ref	1.22 (0.99–1.50)	0.06	1.19 (0.96–1.48)	0.11
Interpersonal	Ref	1.42 (1.11–1.82)	0.005	1.37 (1.10–1.76)	0.01
Existential	Ref	1.17 (0.95–1.43)	0.13	1.14 (0.93–1.40)	0.2
MI domains					
Analytical	Ref	1.23 (0.98–1.56)	0.08	1.21 (0.94–1.53)	0.12
Interactive	Ref	1.35 (1.07–1.71)	0.01	1.36 (1.07–1.73)	0.01
Introspective	Ref	1.29 (1.03–1.63)	0.03	1.26 (0.99–1.59)	0.06
Total MI	Ref	1.33 (1.04–1.69)	0.02	1.31 (1.02–1.67)	0.035

Note: Data were presented as Odds Ratio (95% confidence interval) for MI scores in girls vs. boys. The ORs were adjusted with age and BMI in the adjusted model. *p* < 0.05 was taken as significant.

**Table 3:** Bivariate correlation analysis of serum iron and transferrin saturation levels with other parameters.

Parameters	Iron			TSAT		
	All	Boys	Females	All	Boys	Females
Age	−0.1	0.03	−0.22 **	−0.01	0.09	−0.08
BMI	−0.11 *	−0.1	−0.14 *	0.02	0.08	−0.01
Waist	−0.13 **	−0.05	−0.19 **	0.07	0.19 *	−0.13
Hips	−0.18 **	−0.04	−0.23 **	0.13 *	0.20 *	0.01
WHR	0.07	0.06	−0.03			
Systolic BP	−0.05	−0.02	−0.07	0.09	0.19 *	0.01
Diastolic BP	−0.05	0.04	0.04	0.08	0.19 *	−0.02
Glucose	0.01	−0.1	0.02	0.14 *	0.16	0.23 *
T-Cholesterol	0.16 **	0.13	0.21 **	0.09	0.07	0.11
HDL-Cholesterol (mmol/L)	−0.05	−0.06	−0.06	0.07	−0.06	0.16
Triglycerides	0.28 **	0.26 **	0.27 **	0.12	0.18 *	0.09
MI categories						
Logical	−0.03	0.07	−0.02	0.04	0.16	−0.09
Musical	0.04	−0.01	0.07	0.06	0.12	−0.01
Naturalist	−0.01	0.02	0.01	0.05	0.14	−0.05
Linguistic	−0.04	0.02	−0.03	0.04	0.18 *	−0.11
Social	−0.03	0.01	0.01	0.05	0.16	−0.06
Kinesthetic	−0.05	0.02	−0.1	0.06	0.21 *	−0.1
Spatial	−0.02	0.01	0.01	0.05	0.17	−0.07
Interpersonal	0.01	0.01	0.1	0.03	0.12	−0.09
Existential	0.01	0.04	0.03	0.06	0.22 *	−0.09
MI Domains						
Analytical	0.01	0.03	0.02	0.05	0.15	−0.05
Interactive	−0.04	0.02	−0.03	0.05	0.19 *	−0.1
Introspective	−0.01	0.02	0.03	0.05	0.18 *	−0.09
Total MI	−0.02	0.02	0.01	0.05	0.18 *	−0.08

Note: Data presented as correlation coefficient (r), * denotes significance at the 0.05 level and ** denotes significance at the 0.01 level.

**Table 4:** Mixed-effect regression analysis for serum iron levels with MI scores.

	Unadjusted		Adjusted for Age and BMI	
MI Categories	β (95%CI)	*p*	β (95%CI)	*p*
Logical	−0.15 (−0.9, 0.6)	0.70	−0.08 (−0.9, 0.7)	0.84
Musical	0.26 (−0.5, 1.0)	0.47	0.38 (−0.3, 1.1)	0.30
Naturalist	−0.03 (−0.8, 0.8)	0.94	0.05 (−0.8, 0.9)	0.88
Linguistic	−0.18 (−1, 0.7)	0.66	−0.04 (−0.9, 0.8)	0.92
Social	−0.12 (−1, 0.7)	0.78	−0.004 (−0.8, 0.8)	0.99
Kinesthetic	−0.27 (−1, 0.5)	0.49	−0.15 (−0.9, 0.6)	0.70
Spatial	−0.09 (−0.9, 0.7)	0.83	0.05 (−0.7, 0.8)	0.89
Interpersonal	0.21 (−0.7, 1.1)	0.64	0.22 (−0.7, 1.1)	0.62
Existential	0.05 (−0.7, 0.8)	0.89	0.12 (−0.6, 0.9)	0.75
MI domains				
Analytical	0.06 (−0.8, 0.9)	0.89	0.17 (−0.7, 1)	0.69
Interactive	−0.22 (−1.1, 0.6)	0.61	−0.08 (−1, 0.8)	0.86
Introspective	0.05 (−0.8, 0.9)	0.91	0.14 (−0.7, 1.0)	0.74
Total MI	−0.06 (−0.9, 0.8)	0.89	0.06 (−0.8, 1.0)	0.89

Note: Data presented as β-coefficient and its 95% confidence intervals.

**Table 5:** Regression analysis for MI scores according to transferrin saturation levels (>20% vs. ≤20%).

MI Categories	TSAT ≤ 20%	TSAT > 20%											
		All Subjects (434)				Boys (174)				Girls (260)			
		Unadjusted		Adjusted for Age and BMI		Unadjusted		Adjusted for Age and BMI		Unadjusted		Adjusted for Age and BMI	
		OR (95% CI)	*p*	OR (95% CI)	*p*	OR (95% CI)	*p*	OR (95% CI)	*p*	OR (95% CI)	*p*	OR (95% CI)	*p*
Logical	Ref	1.01 (0.8, 1.3)	0.92	1.03 (0.8, 1.3)	0.82	1.39 (0.9, 2.1)	0.081	1.44 (0.9, 2.1)	0.06	0.71 (0.5, 1.1)	0.10	0.72 (0.5, 1.1)	0.72
Musical	Ref	1.25 (0.7, 1.6)	0.07	1.27 (0.9, 1.6)	0.05	1.52 (1.1, 2.1)	0.011	1.59 (1.1, 2.2)	0.006	0.93 (0.6, 1.4)	0.73	0.94 (0.6, 1.4)	0.77
Naturalist	Ref	1.07 (0.8, 1.4)	0.60	1.09 (0.8, 1.4)	0.52	1.32 (0.9, 1.8)	0.115	1.36 (0.9, 1.9)	0.08	0.81 (0.5, 1.2)	0.80	0.82 (0.5, 1.3)	0.38
Linguistic	Ref	1.08 (0.8, 1.4)	0.57	1.11 (0.8, 1.4)	0.50	1.51 (1.1, 2.2)	0.024	1.57 (1.1, 2.3)	0.02	0.66 (0.4, 1.1)	0.66	0.68 (0.4, 1.1)	0.10
Social	Ref	1.11 (0.9, 1.4)	0.42	1.14 (0.9, 1.5)	0.35	1.39 (0.9, 2.1)	0.076	1.44 (0.9, 2.1)	0.06	0.86 (0.6, 1.3)	0.86	0.89 (0.6, 1.4)	0.60
Kinesthetic	Ref	1.14 (0.9, 1.5)	0.30	1.16 (0.9, 1.5)	0.25	1.45 (1.1, 2.1)	0.033	1.48 (1.1, 2.1)	0.02	0.84 (0.6, 1.2)	0.39	0.87 (0.6, 1.3)	0.51
Spatial	Ref	1.11 (0.9, 1.4)	0.45	1.12 (0.9, 1.4)	0.37	1.38 (0.9, 1.9)	0.056	1.45 (1.1, 2.1)	0.03	0.81 (0.5, 1.2)	0.30	0.84 (0.6, 1.3)	0.39
Interpersonal	Ref	1.03 (0.8, 1.4)	0.84	1.04 (0.8, 1.4)	0.77	1.35 (0.9, 1.9)	0.123	1.39 (0.9, 2.1)	0.09	0.71 (0.4, 1.1)	0.15	0.73 (0.5, 1.2)	0.72
Existential	Ref	1.12 (0.9, 1.4)	0.37	1.13 (0.9, 1.4)	0.32	1.49 (1.1, 2.1)	0.021	1.56 (1.1, 2.2)	0.01	0.77 (0.5, 1.1)	0.20	0.79 (0.5, 1.2)	0.26
MI domains													
Analytical	Ref	1.13 (0.9, 1.5)	0.36	1.16 (0.9, 1.5)	0.30	1.49 (1.1, 2.2)	0.032	1.56 (1.1, 2.3)	0.02	0.77 (0.5, 1.2)	0.27	0.81 (0.5, 1.3)	0.34
Interactive	Ref	1.12 (0.9, 1.5)	0.40	1.14 (0.9, 1.5)	0.34	1.51 (1.1, 2.2)	0.032	1.55 (1.1, 2.3)	0.02	0.77 (0.5, 1.2)	0.24	0.79 (0.5, 1.3)	0.33
Introspective	Ref	1.09 (0.8, 1.4)	0.50	1.11 (0.9, 1.5)	0.43	1.46 (1.1, 2.1)	0.043	1.52 (1.1, 2.2)	0.03	0.74 (0.5, 1.2)	0.18	0.76 (0.5, 1.2)	0.24
Total MI	Ref	1.12 (0.9, 1.5)	0.41	1.14 (0.9, 1.5)	0.34	1.51 (1.1, 2.2)	0.032	1.57 (1.1, 2.3)	0.02	0.75 (0.5, 1.2)	0.22	0.78 (0.5, 1.2)	0.29

Note: Data were presented as Odds Ratio (95% confidence interval) for MI scores in those with TSAT > 20% vs. TSAT ≤ 20%. The OR’s were adjusted with age and BMI in the adjusted model. *p* < 0.05 was taken as significant. The odds of having higher MI scores in individuals with TSAT > 20% vs. TSAT ≤ 20% is depicted as a forest plot in Figure 3.

## Data Availability

Data are included in the article/Supplementary Materials, further inquiries can be directed to the corresponding author.

## Supplementary Materials

The following supporting information can be downloaded at https://www.mdpi.com/article/10.3390/biomedicines12071578/s1. File S1: Arabic version of McKenzie Multiple Intelligence questionnaire; Figure S1: The nine facets and three domains of MI.
