# Supplementary material for: Associations between Serum Iron Indices and Self-Assessed Multiple Intelligence Scores among Adolescents in Riyadh, Saudi Arabia
Source: Biomedicines. 2024 Jul 16;12(7):1578. doi: 10.3390/biomedicines12071578 (PMC11274694; doi:10.3390/biomedicines12071578)
Supplement: Supplementary file 1 [file biomedicines-12-01578-s001.zip › File S1.pdf]

استبانة الذكاءات المتعددة للطلاب

قائمة ماكينزي للذكاءات المتعددة

البيانات الأولية:

اسم الطالب		العمر	
المدرسة		المرحلة الدراسية	

الأبناء الأعزاء **سلمكم الله**

السلام عليكم ورحمة الله وبركاته

السلام عليكم ورحمة الله وبركاته، تهدف هذه الاستبانة إلى قياس الذكاءات المتعددة لديكم، فنأمل التكرم بالتعاون معنا بالاستجابة على كل فقرات الاستبانة، مع تحري الدقة واختيار الاستجابة التي تجدها أقرب لك.

ثانياً: ضع علامة (√) أمام كل عبارة وأسفل التدريج الذي ستجد أنه أقرب لك.

م	العبارة	تنطبق علي تماماً	تنطبق علي كثيراً	تنطبق علي حياناً	تنطبق علي قليلاً	لا تنطبق علي إطلاقاً
1.	أستمتع بقراءة كل أنواع المواد الدراسية					
2.	أحتفظ بأفكاري دقيقة ومرتبة					
3.	أستطيع تخيل الكثير من الأفكار في رأسي					
4.	أستمتع بالعمل اليدوي في الأنشطة الحرفية مثل النجارة والزخرفة					
5.	أعرف المقامات للعديد من المقطوعات النغمية					
6.	أحافظ على معتقداتي الأخلاقية					
7.	أتعلم أفضل من تفاعلي مع الآخرين					
8.	أستمتع بتصنيف الأشياء في مجموعات متجانسة وفقاً لخصائصها					
9.	مهم لي رؤية دوري في صورة كبيرة وسط الآخرين					
10.	أسجل ملاحظات تساعدني على الفهم والتذكر					
11.	أفضل التتابع المنطقي أو السير خطوة —خطوة في فهم الأشياء					
12.	أستمتع بإعادة ترتيب حجرتي بشكل مستمر					
13.	أستمتع بالحركة والنشاط المستمر					
14.	أركز في الأصوات الغنائية والألحان الغنائية					

م	العبارة	تنطبق علي تماماً	تنطبق علي كثيراً	تنطبق علي حياناً	تنطبق علي قليلاً	لا تنطبق علي إطلاقاً
15.	أتعلم أفضل عندما يكون لدى ارتباط عاطفي بالموضوع					
16.	تتسم حياتي بالمرح والتفاؤل					
17.	أهتم بالقضايا البيئية في الأماكن المختلفة					
18.	أستمتع بمناقشة أسئلة حول الحياة					
19.	أتبادل الرسائل مع أصدقائي من خلال وسائل التواصل الاجتماعي					
20.	أستطيع حل المسائل الرياضية بسهولة					
21.	أستمتع بالفنون الإبداعية لاستخدامها ألوانا مًتعددة					
22.	أستمتع بالألعاب الرياضية في الهواء الطلق					
23.	أستطيع أداء بعض الحركات وفق نغمة ما لمقطوعة موسيقية					
24.	أحدد هدفي في الحياة وأفكر فيه بانتظام					
25.	أستمتع بوجودي ضمن مجموعات دراسية منتجة					
26.	أستمتع بالسفر والتجوال وإقامة المخيمات					
27.	أقضى أوقاتاً كثيرة أتأمل في الكون					
28.	من السهل على توضيح أفكاري للآخرين					
29.	أحب التعامل مع الأشخاص المنظمين والمنطقيين					
30.	أتذكر بسهولة الأشياء المنظمة في رسومات وخرائط					
31.	استخدم المهارات الجسمية كلغة إشارة للاتصال بالآخرين					
32.	أهتم بالعزف على آلة موسيقية					
33.	اتجاهاتي لها تأثير على تعلمي في المواقف المختلفة					
34.	أستمتع بغرف الدردشة على الانترنت					
35.	أستمتع بالعمل في الحدائق					
36.	أستمتع بمشاهدة القطع الفنية النادرة					
37.	مهم لي أن أشترك في المجالات المختلفة					
38.	أستطع إنجاز كثير من الحسابات بسرعة في رأسي					
39.	أستمتع بالفنون والأداءات الحركية المختلفة					
40.	أعتقد أن الجسم السليم مهم للعقل السليم					
41.	يجذبني الشعر المنتظم في قافية واحدة					
42.	أهتم بقضية العدالة الاجتماعية بين الأشخاص					

م	العبارة	تنطبق علي تماماً	تنطبق علي كثيراً	تنطبق على حياناً	تنطبق علي قليلاً	لا تنطبق علي إطلاقاً
.43	إبداء الرأي والمشاركة الاجتماعية مهمة لي					
.44	أحافظ على الحدائق العامة لأنها من حق المجتمع					
.45	أستمتع بتدريبات التأمل والاسترخاء					
.46	أستمتع بألغاز الكلمات المتقاطعة الصعبة و المحيرة					
.47	أحب الألغاز التي تتطلب التفكير الاستنتاجي					
.48	أحاول تنظيم الأشياء في مخططات ورسوم بيانية					
.49	تعد الفنون والحرف المختلفة تسالي ممتعة لي					
.50	أتذكر بسهولة الأشياء الموجودة في قافية موسيقية محددة					
.51	عندما أعمل بمفردي أنتج أفضل عن العمل في مجموعة					
.52	أستمتع بالبرامج الحوارية التلفزيونية والإذاعية					
.53	أفضل تنظيم الأشياء في أشكال هرمية عند عرضها					
.54	أحب زيارة الأماكن المدهشة في الطبيعة					
.55	أستمتع بكتابة مذكراتي					
.56	عندما أبدأ مهمة أستطيع الإجابة على كل أسئلتها					
.57	أستمتع بالألعاب ثلاثية الأبعاد					
.58	أستمتع بالتعبيرات الحركية					
.59	أركز في أعمالي أثناء استماعي للراديو والتلفزيون					
.60	احتاج إلى معرفة كل شيء قبل الموافقة على القيام بعمل ما					
.61	أحب العمل في فريق					
.62	أحب جميع أنواع الحيوانات					
.63	أستمتع بالقراءة عن الفلاسفة القدماء والمعاصرين					
.64	اهتم بالتلاعب بالألفاظ والألغاز اللغوية					
.65	اهتم بربط الأشياء ببعضها ربطاً منطقياً وإيجاد العلاقات بينها					
.66	أهتم بصور الأماكن المختلفة واحتفظ بها					
.67	أحب استخدام الأدوات المختلفة في أعمالي					
.68	أستمتع بالعديد من أنواع الغناء					
.69	عندما أثق في الآخرين أعطيهم أكبر قدر من مجهودي					
.70	أشعر بالارتياح عندما أكون وسط مجموعة من الأشخاص					

لا تنطبق علي إطلاقاً	تنطبق علي قليلاً	تنطبق على حياناً	تنطبق علي كثيراً	تنطبق علي تماماً	العبارة	م
					أستمتع باتباع نظام محدد في بيتي	.71
					يسهل تعلمي للأشياء الجديدة عندما أفهم قيمتها	.72
					أهتم باللغات الأجنبية وأحاول تعلمها	.73
					أحب الأعمال على جداول البيانات الحاسوبية وقواعد البيانات	.74
					أستطيع تذكر الأشياء في صور عقلية	.75
					أعيش أسلوب حياة نشطاً وجاداً	.76
					أهتم بالمسرحيات الغنائية أكثر من المسرحيات الأخرى	.77
					أحب أن أكون سبباً في مساعدة الآخرين	.78
					أحب المشاركة في النوادي والأنشطة الثقافية	.79
					أستمتع بدراسة علوم الأحياء والنبات والحيوان	.80
					تعجبني الأشياء الذكية الأخرى في الحياة أو الكون	.81
					أحب المشاركة في الحوارات والمناقشات والخطابة	.82
					اعتقد أن كل شيء له تفسير منطقي مقبول	.83
					أستمتع بقراءة المخططات والخرائط	.84
					أتعلم أفضل من خلال العمل	.85
					أتذكر القصائد الغنائية بسهولة	.86
					أقوم بتصحيح مفاهيم خاطئة لدى الآخرين	.87
					أهتم بالقضايا الاجتماعية ومسبباتها	.88
					أستمتع بقضاء الكثير من الوقت في الهواء الطلق	.89
					أهتم بدراسة التاريخ والثقافة القديمة لتكوين رؤية عن الحياة	.90

Association between Circulating Levels of Iron and Self-assessed Multiple Intelligence in Saudi Adolescents

لعلاقة بين مستوي الحديد في الدم ومستوي ذكاء المراهقين السعوديين باستخدام استبيان التقييم الذاتي للذكاء المتعدد

PI: Ms. Hiba Abdul Qadir Farhan
CO-PI 1: Prof. Nasser Al-Daghri
Co-PI 2: Dr. Shaun Sabico

Section 4: Multiple Intelligence Questionnaire

Serial No: ________ Date: / / 20

Instructions: Please ✓ what best describes you.

1=Strongly disagree, 2=Disagree, 3=Neutral, 4=Agree, 5= Strongly agree.

MI		Questions	1	2	3	4	5
Linguistic	Q1	Foreign languages interest me					
Logical	Q2	I am known for being neat and orderly					
Spatial	Q3	Rearranging a room and redecorating are fun for me					
Kinesthetic	Q4	I learn by doing					
Musical	Q5	I easily pick up on patterns					
Social	Q6	My attitude effects how I learn					
Existential	Q7	It is important to see my role in the “big picture” of things					
Naturalist	Q8	I enjoy categorizing things by common traits					
Interpersonal	Q9	I learn best interacting with others					
Linguistic	Q10	I enjoy reading books, magazines and web sites					
Logical	Q11	Step-by-step directions are a big help					
Spatial	Q12	I enjoy creating my own works of art					

Kinesthetic	Q13	I enjoy making things with my hands					
Musical	Q14	I focus in on noise and sounds					
Social	Q15	I like to be involved in causes that help others					
Existential	Q16	I enjoy discussing questions about life					
Naturalist	Q17	Ecological issues are important to me					
Interpersonal	Q18	I enjoy informal chat and serious discussion					
Linguistic	Q19	I keep a journal					
Logical	Q20	Problem solving comes easily to me					
Spatial	Q21	I remember better using graphic organizers					
Kinesthetic	Q22	Sports are a part of my life					
Musical	Q23	Moving to a beat is easy for me					
Social	Q24	I am keenly aware of my moral beliefs					
Existential	Q25	Religion is important to me					
Naturalist	Q26	Classification helps me make sense of new data					
Interpersonal	Q27	The more the merrier					
Linguistic	Q28	Word puzzles like crosswords or jumbles are enjoyable					
Logical	Q29	I get easily frustrated with disorganized people					
Spatial	Q30	I enjoy all kinds of entertainment media					
Kinesthetic	Q31	I use gestures and non-verbal cues when I communicate					
Musical	Q32	I enjoy making music					
Social	Q33	I learn best when I have an emotional attachment to the subject					
Existential	Q34	I enjoy viewing art work					
Naturalist	Q35	I enjoy working in a garden					
Interpersonal	Q36	I often serve as a leader among peers and colleagues					
Linguistic	Q37	Taking notes helps me remember and understand					
Logical	Q38	I can complete calculations quickly in my head					
Spatial	Q39	Charts, graphs and tables help me interpret data					

Kinesthetic	Q40	Demonstrating is better than explaining					
Musical	Q41	I respond to the cadence of poetry					
Social	Q42	Fairness is important to me					
Existential	Q43	Relaxation and meditation exercises are rewarding to me					
Naturalist	Q44	I believe preserving our National Parks is important					
Interpersonal	Q45	I value relationships more than ideas or accomplishments					
Linguistic	Q46	I faithfully contact friends through letters and/or e-mail					
Logical	Q47	Logic puzzles are fun					
Spatial	Q48	A music video can make me more interested in a song					
Kinesthetic	Q49	I love to dance					
Musical	Q50	I remember things by putting them in a rhyme					
Social	Q51	Social justice issues interest me					
Existential	Q52	I like traveling to visit inspiring places					
Naturalist	Q53	Putting things in hierarchies makes sense to me					
Interpersonal	Q54	Study groups are very productive for me					
Linguistic	Q55	It is easy for me to explain my ideas to others					
Logical	Q56	I can't begin an assignment until I have all my "ducks in a row"					
Spatial	Q57	I can recall things as mental pictures					
Kinesthetic	Q58	I like working with tools					
Musical	Q59	Concentration is difficult for me if there is background noise					
Social	Q60	Working alone can be just as productive as working in a group					
Existential	Q61	I enjoy reading philosophers					
Naturalist	Q62	Animals are important in my life					
Interpersonal	Q63	I am a “team player”					
Linguistic	Q64	I write for pleasure					
Logical	Q65	Structure is a good thing					
Spatial	Q66	I am good at reading maps and blueprints					

Kinesthetic	Q67	Inactivity can make me more tired than being very busy					
Musical	Q68	Listening to sounds in nature can be very relaxing					
Social	Q69	I need to know why I should do something before I agree to do it					
Existential	Q70	Learning new things is easier when I see their real world application					
Naturalist	Q71	My home has a recycling system in place					
Interpersonal	Q72	Friends are important to me					
Linguistic	Q73	Puns, anagrams and spoonerisms are fun					
Logical	Q74	I enjoy troubleshooting something that isn't working properly					
Spatial	Q75	Three dimensional puzzles are fun					
Kinesthetic	Q76	Hands-on activities are fun					
Musical	Q77	Musicals are more engaging to me than dramatic plays					
Social	Q78	When I believe in something I give more effort towards it					
Existential	Q79	I wonder if there are other forms of intelligent life in the universe					
Naturalist	Q80	I enjoy studying biology, botany and/or zoology					
Interpersonal	Q81	I belong to more than three clubs or organizations					
Linguistic	Q82	I enjoy public speaking and participating in debates					
Logical	Q83	Things have to make sense to me or I am dissatisfied					
Spatial	Q84	I can visualize ideas in my mind					
Kinesthetic	Q85	I live an active lifestyle					
Musical	Q86	Remembering song lyrics is easy for me					
Social	Q87	I am willing to protest or sign a petition to right a wrong					
Existential	Q88	It is important for me to feel connected to people, ideas and beliefs					
Naturalist	Q89	I pick up on subtle differences in meaning					
Interpersonal	Q90	I dislike working alone					

Note: The responses of the 10 questions from each of the MI category will be used to generate the average values for each participant.
