# Supplementary figures and images for: Associations between Serum Iron Indices and Self-Assessed Multiple Intelligence Scores among Adolescents in Riyadh, Saudi Arabia
Source: Biomedicines. 2024 Jul 16;12(7):1578. doi: 10.3390/biomedicines12071578 (PMC11274694; doi:10.3390/biomedicines12071578)

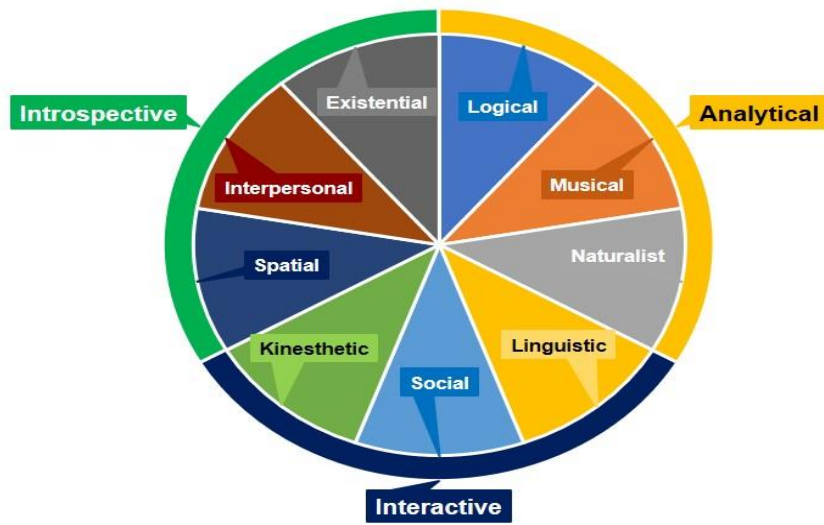

**Figure S1:** The nine facets and three domains of MI.

Supplement: Supplementary file 1 [file biomedicines-12-01578-s001.zip › Figure S1.pdf]
